# Systemic AAVrh10 provides higher transgene expression than AAV9 in the brain and the spinal cord of neonatal mice

**DOI:** 10.3389/fnmol.2015.00036

**Published:** 2015-07-28

**Authors:** Yannick Tanguy, Maria G. Biferi, Aurore Besse, Stephanie Astord, Mathilde Cohen-Tannoudji, Thibaut Marais, Martine Barkats

**Affiliations:** Center of Research on Myology, FRE 3617 Centre National de la Recherche Scientifique, UMRS 974 INSERM, French Institute of Myology, Pierre and Marie Curie UniversityParis, France

**Keywords:** adeno-associated virus, AAV9, AAVrh10, gene therapy, central nervous system, peripheral nervous system, motor neuron, SMA

## Abstract

Systemic delivery of self-complementary (sc) adeno-associated-virus vector of serotype 9 (AAV9) was recently shown to provide robust and widespread gene transfer to the central nervous system (CNS), opening new avenues for practical, and non-invasive gene therapy of neurological diseases. More recently, AAV of serotype rh10 (AAVrh10) was also found highly efficient to mediate CNS transduction after intravenous administration in mice. However, only a few studies compared AAV9 and AAVrh10 efficiencies, particularly in the spinal cord. In this study, we compared the transduction capabilities of AAV9 and AAVrh10 in the brain, the spinal cord, and the peripheral nervous system (PNS) after intravenous delivery in neonatal mice. As reported in previous studies, AAVrh10 achieved either similar or higher transduction than AAV9 in all the examined brain regions. The superiority of AAVrh10 over AAV9 appeared statistically significant only in the medulla and the cerebellum, but a clear trend was also observed in other structures like the hippocampus or the cortex. In contrast to previous studies, we found that AAVrh10 was more efficient than AAV9 for transduction of the dorsal spinal cord and the lower motor neurons (MNs). However, differences between the two serotypes appeared mainly significant at low dose, and surprisingly, increasing the dose did not improve AAVrh10 distribution in the spinal cord, in contrary to AAV9. Similar dose-related differences between transduction efficiency of the two serotypes were also observed in the sciatic nerve. These findings suggest differences in the transduction mechanisms of these two serotypes, which both hold great promise for gene therapy of neurological diseases.

## Introduction

Nervous system diseases, including functional and degenerative disorders, can affect all cell types in the central (CNS) and peripheral (PNS) nervous system, leading to severe disabilities and patient death in the most severe cases. Due to their devastating consequences, lack of efficient treatments, and aging of the population, these pathologies are becoming a major concern for public health. Advances in molecular technologies have allowed emergence and rapid progress of gene therapy for high and sustained expression of therapeutic proteins in nervous cells. Particular effort has been focused on vector expression and delivery systems, those derived from the adeno-associated virus (AAV) appearing as one of the most promising for gene therapy of nervous diseases (Weinberg et al., 2013).

AAV vectors are non-pathogenic and capable of transducing non-dividing cells permanently, with no toxicity or significant immune reaction (McCown et al., 1996; Wu et al., 1998). A number of Phase I and Phase II clinical trials utilizing AAV vectors have been carried out worldwide (Grieger and Samulski, 2012), and among them, direct injection into the nervous parenchyma of patients with neurological diseases has shown its efficacy and excellent safety profile in several previous clinical trials (Mandel and Burger, 2004; Kaplitt et al., 2007; Marks et al., 2008; Christine et al., 2009; LeWitt et al., 2011; Tardieu et al., 2014). However, due to the impermeability nature of the blood-brain-barrier (BBB), systemic gene transfer to the CNS has been particularly challenging, whether with AAV or any other gene vector. A significant breakthrough has been made in 2007, with our discovery that self-complementary serotype 9 AAV vectors (scAAV9) are capable to achieve widespread gene transfer to the CNS after systemic delivery (Barkats, 2007). Although transgene expression has been firstly reported to be primarily restricted to astrocytes after intravenous (IV) injection in adult mice (Foust et al., 2009), we showed that systemic delivery of an AAV9 encoding the green-fluorescent-protein (GFP) mediated efficient transduction of a relatively large proportion of neurons in adult mice (Duqué et al., 2009). The comparison of single-stranded and self-complementary AAV of serotype 1 and 9 for transduction of the mouse CNS after IV delivery showed indeed that self-complementary AAV9 was the most efficient vector for transducing spinal cord cells including motor neurons (MNs), and that transgene expression lasted at least 5 months (the duration of the study) (Duqué et al., 2009). Importantly, this finding was successfully translated to a domestic cat strain with deletions of the LIX1 gene (Fyfe et al., 2006), a model of autosomal recessive spinal muscular atrophy (SMA) similar to human type III SMA (Duqué et al., 2009). The remarkable potential of systemic AAV9 for transducing MNs in adult animals was further confirmed in both rodents and large animals including non-human primates (NHPs) (Bevan et al., 2011; Gray et al., 2011). In addition to these IV studies, we recently reported that intramuscular (IM) delivery of AAV9 was also effective to achieve widespread gene transfer to the CNS in both neonatal and adult mice. Indeed, AAV9 delivery into the gastrocnemius muscle mediated gene transfer not only into the lumbar MNs, but also at the upper levels of the spinal cord and in discrete parts of the brain (Benkhelifa-Ziyyat et al., 2013). Importantly, either IV or IM delivery of AAV9 vectors engineered to overexpress the *Survival of Motor Neuron gene 1* (*SMN1*) gene dramatically rescued the pathological phenotype in a mouse model of spinal muscular atrophy (SMA) (Foust et al., 2010; Valori et al., 2010; Dominguez et al., 2011; Benkhelifa-Ziyyat et al., 2013). In particular, we found that a single IV delivery of an optimized *SMN1*-encoding AAV9 vector (AAV9-SMN1opti) in neonatal SMNΔ7 mice, a mouse model of human SMA (Le et al., 2005), increased life expectancy up to 355 days in mice that normally survive about 13 days (Dominguez et al., 2011). The AAV9-SMN1opti treatment also partially corrected the body weight loss phenotype, improved motor activity, and prevented MN degeneration (Dominguez et al., 2011). Systemic AAV9 delivery was further shown to be very promising for treating other neurological or lysosomal diseases, including amyotrophic lateral sclerosis (Yamashita et al., 2013), Canavan disease (Ahmed et al., 2013) or MPSIIIA (Fu et al., 2011; Ruzo et al., 2012), highlighting the outstanding potential of this approach for a large range of CNS and systemic pathologies.

Although AAV9 is usually considered as the most promising vector for achieving widespread CNS transduction, alternative AAV vectors with increased spread and transduction efficiency are currently actively investigated. In particular, the AAV of serotype rh10 (AAVrh10), which has been isolated from rhesus monkeys (Gao et al., 2002, 2003), was recently reported to be as least as efficient as AAV9 for transduction of many tissues including the CNS in neonatal mice (Hu et al., 2010; Zhang et al., 2011). In particular, using a scoring system to evaluate GFP-immunoreactivity in different CNS regions, Zhang et al. showed that AAVrh10 transduction efficiency was comparable to that of AAV9 in the spinal cord, and was globally higher than that of AAV9 in the brain (with differences according to the brain region) (Zhang et al., 2011).

In this study, we used semi-quantitative and quantitative analyses to compare the ability of AAV9 and AAVrh10 for achieving gene transfer to the CNS and the PNS following intravascular delivery in neonatal mice. We found that low dose AAVrh10 induced higher transduction than AAV9 of most regions that we examined, in particular the medulla, the cerebellum, the spinal cord and the sciatic nerve. However, differences between the two serotypes were less evident were the vector doses were increased, suggesting serotype-related differences in the transduction process.

## Materials and methods

### Animals

Wild-type animals were obtained from Smn1^+/−^, Smn2^+/+^ breeding (FVB.Cg-Tg(SMN2)89Ahmb Tg (SMN1^*^A2G)2023Ahmb Smn1 tm1Msd/J) (number 5024, Jackson Laboratories, Main Harbor, USA). Mice were housed under controlled conditions (22 ± 2°C, 50 ± 10% relative humidity, 12 h/12 h light/dark cycle, food, and water *ad libitum*). All animal experiments were carried out in accordance with European guidelines for the care and use of experimental animals and approved by the Charles Darwin N°5 Ethics Committee on Animal Experiments (agreement n°01883.02-16/9/14).

### Production of AAV vectors and intravenous delivery

AAV vectors of serotype 9 or rh10, carrying the GFP under the control of the cytomegalovirus immediate/early (CMV) promoter were prepared by the triple transfection method in HEK293T cells, as previously described (Duqué et al., 2009). Briefly, cells were transfected with (i) the adenovirus helper plasmid, (ii) the AAV packaging plasmid encoding the rep2 and cap9 (p5E18-VD2/9) or cap-rh10 genes, and (iii) the AAV2 plasmid expressing CMV-GFP. Seventy-two hours after transfection, cells were harvested, frozen/thawed four times, and AAV vectors were purified by ultracentrifugation through an iodixanol gradient (Sigma-Aldrich, St Quentin Fallavier, France) and concentrated with Amicon Ultra–Ultra cell 100K filter units (Millipore) in PBSMK buffer (0.1 M phosphate buffered saline solution (PBS), 1 mM MgCl_2_ and 2.5 mM KCl). Aliquots were stored at −80°C until use. Vector titers were determined by real-time PCR and expressed as viral genomes per mL (vg/mL).

Neonatal mice (P0) received 40 μL of viral suspension containing 3 × 10^10^ or 1 × 10^11^ vg of AAV9 or AAVrh10 into the temporal vein using an Hamilton syringe with a 32-gauge needle (Hamilton).

### Western blot

Animals were lethally anesthetized and transcardially perfused with 0.1 M PBS. Tissues were immediately frozen in liquid nitrogen and stored at −80°C until use. For protein extraction, tissues were grinded in a lysis buffer (150 mM NaCl, 50 mM Tris–HCl, 0.5% sodium deoxycholate, 1% NP40, 1% SDS) supplied with protease inhibitors cocktail (Complete Mini, Roche Diagnostics). Lysates were quantified with the DC protein assay (BioRad,) and 50 μg were loaded on a 10% polyacrylamide gel (Criterion XT 10% bis-Tris, Biorad). Proteins were transferred onto a PVDF membrane (Imobilon P, Millipore). Successively, membranes were blocked with a Tris-buffered saline solution (10 mM Tris–HCl pH 7.4, 150 mM NaCl) and 0.05% Tween 20 (TBS-T) containing 5% fat-free dry milk. Membranes were incubated overnight at 4°C with a rabbit anti-GFP antibody (1:10,000; Abcam) or a mouse anti-α-tubulin antibody (1:10,000; Sigma-Aldrich) diluted in TBS-T, 5% fat-free dry milk. After washes in TBS-T buffer, membranes were incubated with horseradish peroxidase conjugated anti-mouse or anti-rabbit secondary antibodies (1:10,000, Amersham Pharmacia Biotech) for 1 h at room temperature. Western blots were developed using SuperSignal West Dura kit (Thermoscientific).

### Immunofluorescence

Animals were lethally anesthetized and transcardially perfused with 0.1 M PBS followed by 4% paraformaldehyde (PFA; Sigma-Aldrich) in PBS. Tissues were harvested and successively incubated in 4% PFA (24 h at 4°C) and in a PBS-sucrose solution (30% sucrose for the spinal cord, 15% sucrose for the other organs, overnight at 4°C). Samples were imbedded with optimal cutting temperature medium (Tissue-Tek OCT; Sakura Finetek) and frozen in cold isopentane. Fourteen μm-thick sections were serially cut on a cryostat (Leica Microsystems) and stored at −80°C.

For immunofluorescence staining, sections were incubated in a blocking solution containing 4% donkey serum, 5% Bovine serum albumin in a PBS-triton X-100 buffer (0.1 M PBS, 0.4 % Triton X-100) for 1 h at room temperature. Sections were incubated with primary antibodies: anti-GFP (1:2,000, rabbit; Abcam), anti-Neurofilament (NF, mouse, 1:500; Millipore), anti-β-S100 (rabbit, 1:200; Dako) or anti-Choline Acetyltransferase (ChAT, goat, 1:100, Millipore), in the blocking solution, overnight at 4°C. After PBS washings, sections were incubated with secondary antibodies conjugated with Alexa Fluor 488 (1:500) or 594 (1:300) (Molecular Probes-Invitrogen). Nuclei were counterstained with 4′,6′-diamidino-2-phénylindole (DAPI, 0.5 μg/mL in PBS; Sigma-Aldrich) and mounted with Fluoromount (Southern Biotech). Pictures were obtained with a confocal laser scanning microscope (Leica) or a motorized fluorescence microscope (AxioImager.Z1; Zeiss).

To quantify GFP expression, representative images from each tissue were taken at identical camera and microscope settings with a fluorescence microscope. For every image, the brightness and background values were measured with ImageJ software (Rasband 1997–2006; National Institutes of Health, Bethesda, MD, http://rsb.info.nih.gov/ij/), and the results corresponded to the mean value of 6 (brain and medulla) or 12 (cerebellum, dorsal spinal cord, sciatic nerve, DRG, heart, and liver) images for each mouse.

### Statistics

All data were analyzed using Prism software (version 4.0, GraphPad). A Mann-Whitney test was used for the analysis of western blot results, *t*-test and One-Way ANOVA were performed for analysis of MN counting. Fluorescence imaging data were treated with either a Mann-Whitney or a Two-Way ANOVA test, which were chosen according to the number of samples. Significant levels were noted as follows: ^*^, *p* < 0.05; ^**^, *p* < 0.01; ^***^, *p* < 0.001.

## Results

### Intravenous AAVrh10 provides a similar or higher brain transduction level than AAV9

**Figure 1:**
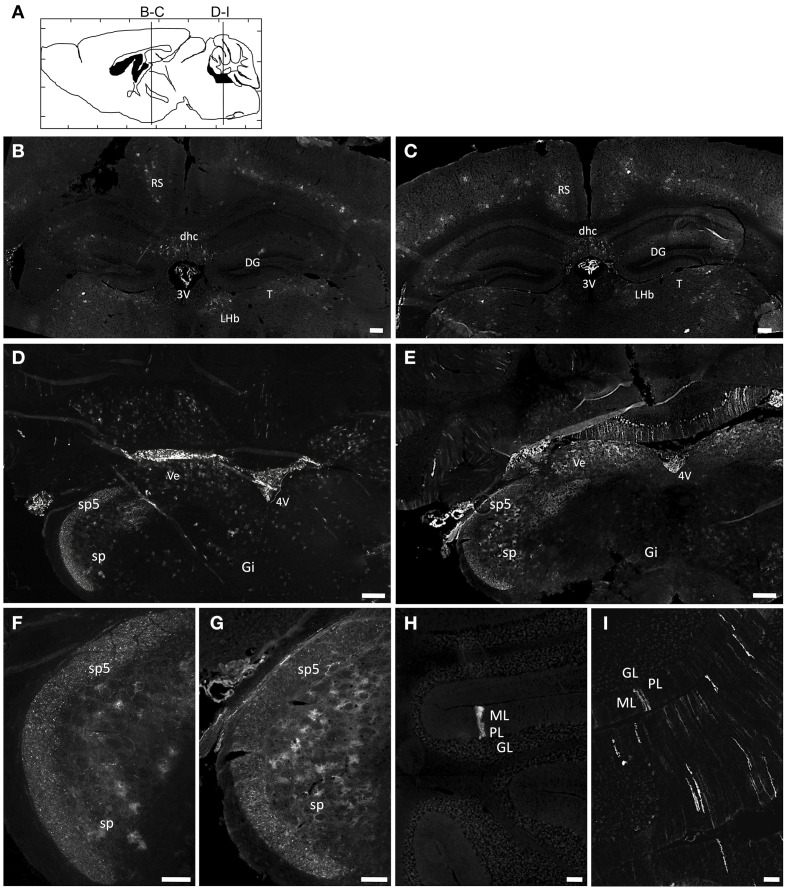
**Immunofluorescence analysis of GFP expression in the brain of AAV9 or AAVrh10 injected mice**. Representative brain sections treated for GFP immunofluorescence 30 days after injection of GFP-expressing AAV9 and AAVrh10 vectors into the facial vein of neonatal mice at P0 (3 × 10^13^ vg/kg, *n* = 4 per group) **(A)** Schematic representation of the investigated areas (**B,C**, Bregma: −1.46 mm; **D–I**, Bregma: −6.48 mm). **(B–I)** Comparison of GFP expression in AAV9-GFP **(B,D,F,H)** or AAVrh10-GFP **(C,E,G,I)** injected mice in **(B,C)** the hippocampus **(D,E)** the medulla **(F,G)** the spinal trigeminal tractus and nucleus, and **(H,I)** the cerebellum. 3V, third Ventricle; 4V, fourth Ventricle; DG, Dentate Gyrus; dhc, dorsal hippocampal commissure; Gi, Gigantocellular reticular nucleus; GL, Granular layer; LHb, Lateral Habenular nucleus; ML, Molecular layer; PL, Purkinje layer; RS, Retrosplenial cortex; sp5, Spinal trigeminal tractus; sp, Spinal trigeminal nucleus; T, Thalamus; Ve, Vestibular nucleus. Scale bars = **(B–E)** 250 μm; **(F–I)** 125 μm.

**Figure 2:**
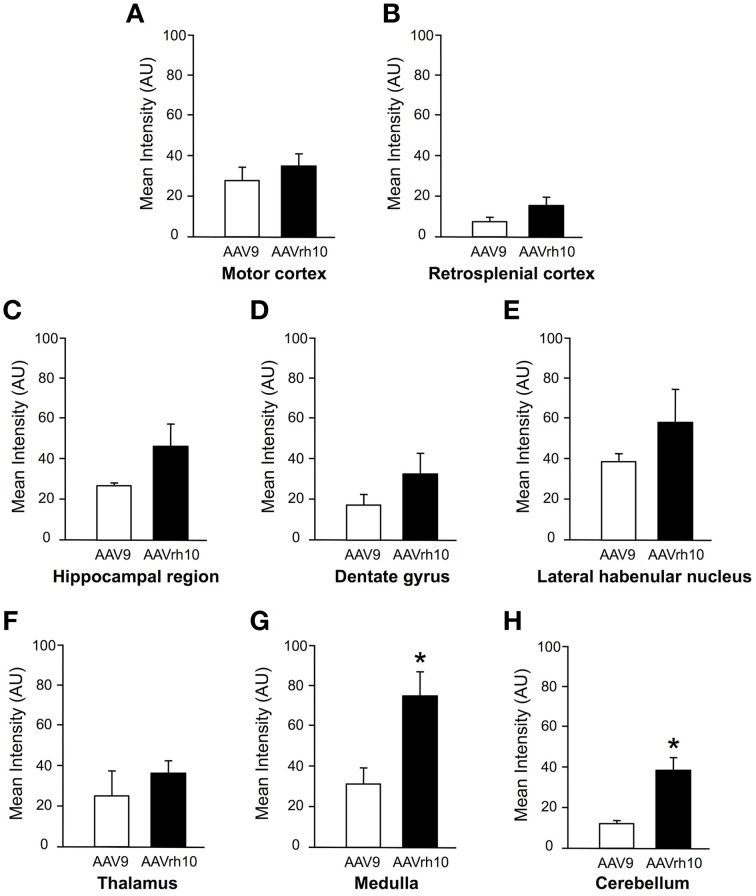
**Quantification of GFP fluorescence intensity in the brain of AAV9 or AAVrh10 injected mice**. Average GFP signal intensity/pixel was measured 30 days after intravenous delivery of AAV9-GFP or AAVrh10-GFP (3 × 10^13^ vg/kg, *n* = 4 for each vector) in neonatal mice. **(A)** Motor cortex **(B)** retrosplenial cortex **(C)** hippocampal region **(D)** dentate gyrus **(E)** lateral habenular nucleus **(F)** thalamus **(G)** medulla **(H)** cerebellum. The data represent the mean values ± SEM of GFP fluorescence intensity/pixel (Mann-Withney test; ^*^p < 0.05).

Newborn mice were injected at P0 into the superficial temporal vein with the AAV9-GFP (*n* = 4) or AAVrh10-GFP (*n* = 4) vectors (3 × 10^13^ vg/kg), and transduction efficiency was first compared in the brain by immunofluorescence analysis 1 month after injection. For both vectors, a gradient of expression was observed from the brain ventricles and adjacent regions (Bregma −1.46 and −6.48 mm), to more distant brain regions (Figure 1). GFP immunostaining was particularly intense in the choroid plexus of the lateral, 3rd and 4th ventricles, and in neighboring structures such as the lateral habenular nucleus, the CA2 field of the hippocampus, the dorsal hippocampal commissure, the deeper layers of the cerebral cortex, the retrosplenial cortex, the vestibular nucleus and the spinal trigeminal nucleus of the medulla, and the lobule 10 of the cerebellum (Figure 1). In contrast, only a few GFP-expressing cells were observed in regions located far from the ventricles, such as the reticular nucleus, the thalamus or the external lobules of the cerebellum. In all the examined regions, a similar or higher level of transduction was observed with AAVrh10 compared to AAV9, AAVrh10 providing the greatest levels of expression in the cerebellar Purkinje cells, the vestibular and spinal trigeminal nuclei of the medulla, the lateral habenular nucleus, and the deep cortical layers (Figure 1). A quantitative analysis of the GFP signal (mean intensity/pixel) in several brain structures confirmed a strong tendency for a superior transduction efficiency of the AAVrh10, however the difference with AAV9 only reached statistical significance for the medulla (30.9 ± 8 vs. 74.2 ± 12.9 for AAV9 and AAVrh10, respectively; *p* = 0.0286) and the cerebellum (11.7 ± 1.7 vs. 38.5 ± 6.1 for AAV9 and AAVrh10, respectively; *p* = 0.0286) (Figure 2).

### GFP expression in the spinal cord of AAV9 and AAVrh10 injected mice

To compare transduction levels provided by the AAV9 and AAVrh10 serotypes in the spinal cord, neonatal mice were injected at birth with the two GFP-expressing vectors. Both vectors were delivered at low (3 × 10^13^ vg/kg, *n* = 6 per AAV) and high dose (10^14^ vg/kg, *n* = 4 for AAV9 and *n* = 3 for AAVrh10) and GFP expression was evaluated 30 days after injection by western blot analysis on spinal cord protein extracts.

**Figure 3:**
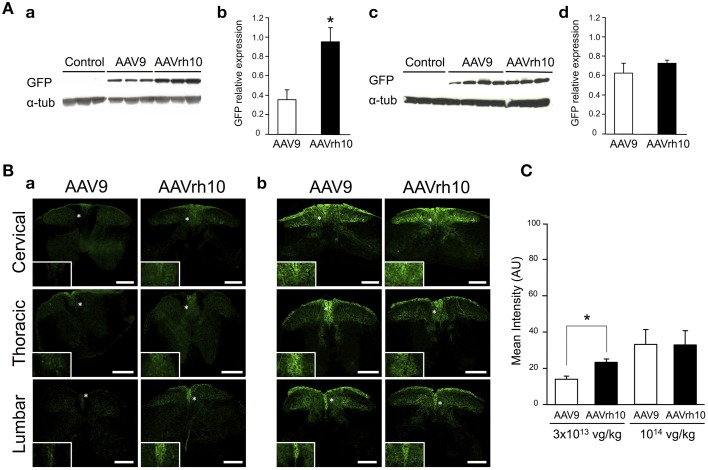
**GFP expression in the spinal cord of AAV9-GFP or AAVrh10-GFP injected mice. (A)** Western blot and **(B)** immunofluorescence analyses were performed 30 days after intravenous injection of the vectors in neonatal mice. **(A)** Western-blot and corresponding densitometric analyses of GFP expression in whole spinal cord lysates injected with (a,b) low dose (3 × 10^13^ vg/kg) or (c,d) high dose (10^14^ vg/kg) of AAV9-GFP or AAVrh10-GFP vectors (*n* = 6 for low dose AAV9 or AAVrh10, *n* = 4 and 3 for high dose AAV9 and AAVrh10, respectively). Controls were protein extracts from non-injected mice and α-tubulin was used as internal loading control. Data in (b,d) represent the mean values ± SEM of GFP levels relative to α-tubulin levels (Mann-Withney test; ^*^p < 0.05). **(B)** Representative sections of the cervical, thoracic and lumbar spinal cord treated for GFP immunofluorescence after injection of AAV9 and AAVrh10 at (a) low dose (3 × 10^13^ vg/kg, *n* = 4 per vector) or (b) high dose (10^14^ vg/kg, *n* = 4 per vector). Panels at the bottom left corner showed high magnification indicated with a white asterisk. Scale bars = 250 μm. **(C)** GFP fluorescence intensity/pixel in the dorsal spinal cord of mice (*n* = 4 per vector and per dose). Results are expressed as mean of four independent experiments ± SEM (Two-Way ANOVA test, Bonferroni Post-test: ^*^p < 0.05).

Similarly to the results in brain, GFP protein levels were found to be increased in spinal cord extracts from mice injected with AAVrh10 compared to AAV9 at 3 × 10^13^ vg/kg (*p* = 0.011) (Figure 3). At this low dose, only a weak GFP expression was observed with both vectors, which was essentially confined to the dorsal part of the spinal cord (corresponding to the sensitive nerves of the fasciculus gracilis and cuneatus) (Figure 3Ba). At the highest dose (10^14^ vg/kg), GFP expression levels were largely increased in the spinal cord, but no statistically significant difference was evidenced between the two vectors (Figure 3). Quantitative analysis of the intensity of GFP immunofluorescence in the dorsal spinal cord columns confirmed the superiority of AAVrh10 vs. AAV9 in this region at low dose (*p* = 0.0157) (Figure 3C).

**Figure 4:**
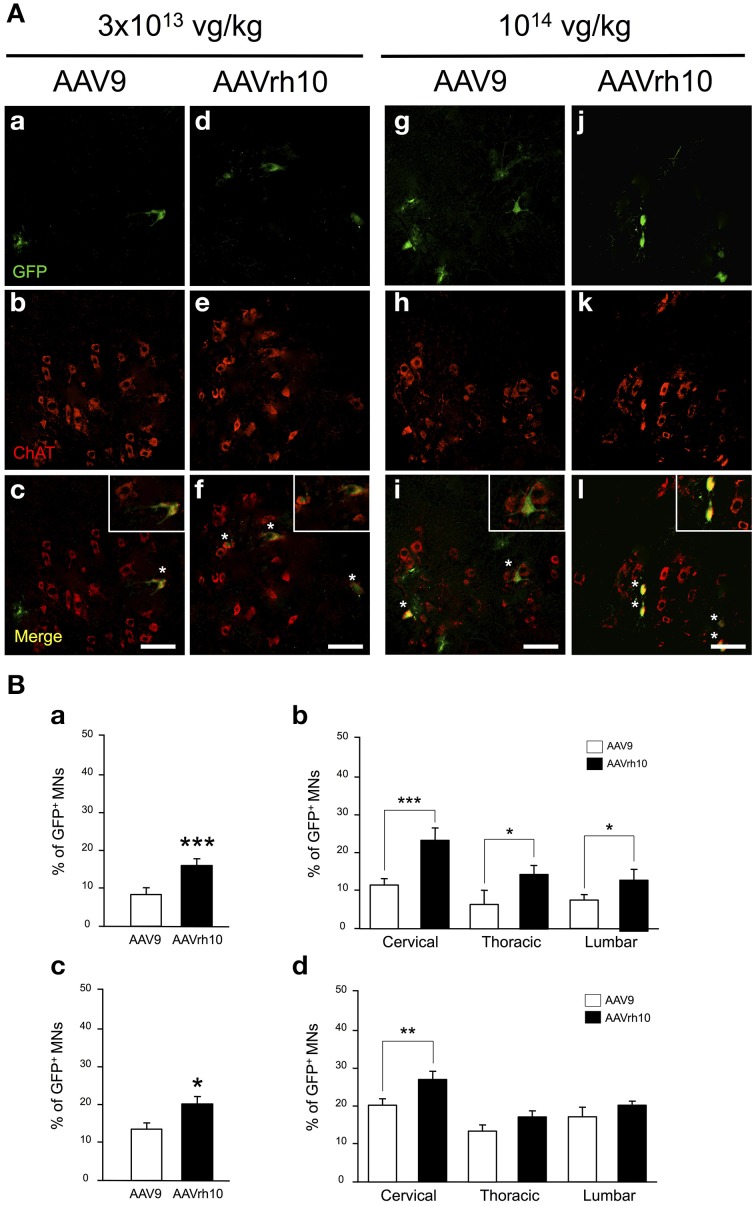
**Comparison of lower motor neuron transduction in AAV9 and AAVrh10 injected mice. (A)** Representative cervical spinal cord sections treated for GFP/ChAT co-immunostaining 30 days after intravenous injection of neonatal mice with low (a–f) and high (g–l) doses of AAV9 or AAVrh10 vectors (a,d,g,j) GFP-positive cells (b,e,h,k) ChAT-positive MNs; (c,f,i,l) merge (asterisks: double-stained MNs; panels at the top right corner: high magnification). Scale bars = 50 μm. **(B)** Percentage of GFP/ChAT-positive MNs in the whole spinal cord (a,c) and in each spinal cord segment (b,d) at low (a,b) and high (c,d) dose of AAV. Data are means ± SEM (*n* = 4) and differences between groups were analyzed by Student *t*-test in (a,c) and One-Way ANOVA, Newman-Keuls *post-hoc*-test in (b,d); ^*^p < 0.05; ^**^p < 0.01; ^***^p < 0.001.

As illustrated in Figure 4, GFP expression was also detected in the ventral horn of the spinal cord, from the cervical to lumbar levels (Figure 4A). MN transduction efficiency mediated by AAV9 and AAVrh10 was compared by co-immunofluorescence analysis using GFP and Choline Acetyltransferase (ChAT), a MN marker. Counting of GFP-positive ChAT MNs revealed that the mean percentage of transduced MNs was doubled throughout the spinal cord from mice injected with AAVrh10 compared to AAV9 at low dose (Figures 4Ba,Bb). Indeed, 22.1, 12.9, and 12.8% of MNs were transduced in the cervical, thoracic, and lumbar spinal cord after injection of low dose AAVrh10, vs. 11.8, 6.16, and 7.26% with AAV9 (*p* = 0.0001, 0.0403, and 0.0318) (Figures 4Ba,Bb). The superiority of AAVrh10 over AAV9 was less striking at the highest dose but was still significant in the whole spinal cord (Figure 4Bc). However, MN transduction analysis in each spinal cord segment showed a statistically significant difference only at the cervical level (25.9 vs. 20.2% for AAVrh10 and AAV9, respectively; *p* = 0.0049) (Figure 4Bd).

### Comparison of AAV9 and AAVrh10-mediated transduction of the peripheral nervous system

**Figure 5:**
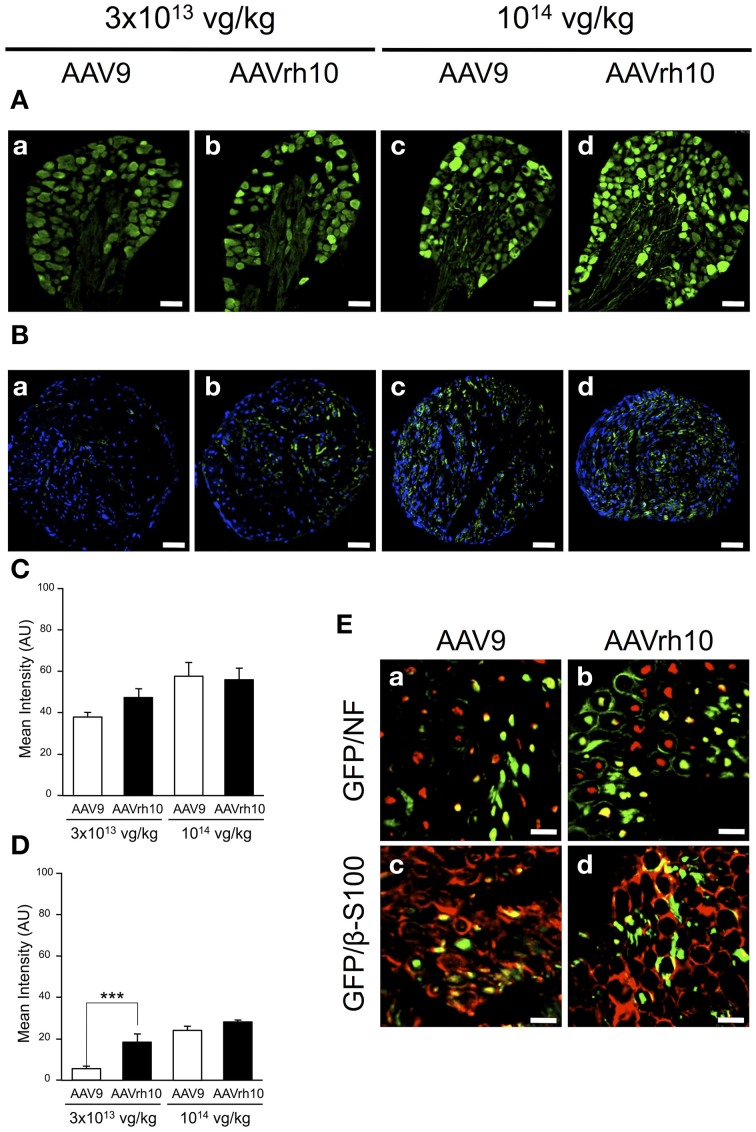
**GFP expression in the dorsal root ganglia and sciatic nerve of AAV9-GFP and AAVrh10-GFP injected mice. (A,B)** Representative sections of **(A)** dorsal root ganglia and **(B)** sciatic nerve, treated for GFP immunofluorescence 30 days after neonatal delivery of AAV9 or AAVrh10 at low (a,b) and high (c,d) dose (*n* = 4 per dose and per serotype) **(C,D)** Quantification of GFP intensity/pixel in the dorsal root ganglia **(C)** and sciatic nerve **(D)** sections. Results are expressed as mean ± SEM (*n* = 4; Two-Way ANOVA analysis, Bonferroni *Post-hoc*-test: ^***^p < 0.001). **(E)** Representative sciatic nerve sections treated for (**a,b**) GFP/NF co-immunostaining (green: GFP; red: NF; yellow: merge) or (c,d) GFP/β-S100 co-immunostaining (green: GFP; red: β-S100; yellow: merge), 30 days after intravenous injection of neonatal mice with AAV9 (a,c) or AAVrh10 (b,d) at high dose (10^14^ vg/kg). Scale bars = **(A)** 50 μm; **(B)** 100 μm; **(C)** 20 μm.

We further compared transgene expression provided by the two AAV serotypes in the PNS, in particular the dorsal root ganglia (DRG) and the sciatic nerve. In the DRG, no significant difference was observed between AAV9 and AAVrh10 injected animals, although a strong tendency for an increased GFP immunofluorescence intensity was noted with AAVrh10 compared to AAV9 at the low dose (Figures 5A,C). Similarly, a difference between the two vectors was observed for transduction of the sciatic nerve only at the low dose (*p* = 0.02) (Figures 5B,D). At high dose, the intensity of GFP immunofluorescence was markedly increased, in particular with AAV9, with no significant difference between the two serotypes (*p* = 0.64) (Figures 5B,D). This dose-related effect on the transduction efficiency provided by AAV9 and AAVrh10 was demonstrated by a statistically significant “AAV serotype by dose” interaction (Two-Way ANOVA, *p* = 0.006).

To further examine the distribution of the GFP protein in the sciatic nerve, co-immunofluorescence analyses were performed using antibodies against neurofilament (NF), a specific axonal marker, and β-S100, a marker for myelinating and non-myelinating Schwann cells. Our results showed that both AAV9 and AAVrh10 provided efficient transduction of NFs (Figures 5Ea,Eb) and Schwann cells (Figures 5Ec,Ed), with no obvious difference between the two serotypes. Of note, GFP was highly expressed in the Schwann cells that surrounded the axons, highlighting gene transfer in the myelinating subpopulation (Figures 5Ec,Ed).

### Comparison of AAVrh10 and AAV9-mediated heart and liver transduction

We finally examined two tissues, the heart and the liver, which were previously demonstrated as differentially transduced following intravascular injection of AAV9 or AAVrh10 (Hu et al., 2010; Piras et al., 2013). A global high transduction of the heart was found for both serotypes, the GFP-positive cells appearing widely distributed in the atria and ventricles (Supplementary Figures 1Aa–d). However, AAVrh10-mediated transduction of the cardiomyocytes was significantly higher than that provided by AAV9 at both low and high doses (Two-Way ANOVA, *p* = 0.0051) (Supplementary Figure 1Ae). The liver was found relatively weakly transduced, in particular at low dose (Supplementary Figures 1Ba–d), but similarly to results in the heart, a tendency for an increased transduction level was found with AAVrh10, although the difference between the two serotypes did not reach any statistical significance (Supplementary Figure 1Be).

## Discussion

A number of comparative studies have reported improved CNS gene transfer after brain injection of AAV9 and AAVrh10, two newly identified AAV serotypes which have been isolated from the tissues of NHPs (rhesus or cynomolgus monkeys) (Gao et al., 2002, 2003; Mori et al., 2004). Both vectors were found to provide more efficient and widespread neuronal transduction in rodents as compared to the first characterized AAV2 serotype, or even to other robust serotypes such as AAV8, with some variability capabilities at spreading and transducing specific brain structures (Cearley and Wolfe, 2006; Sondhi et al., 2007; Klein et al., 2008; Miyake et al., 2011; Rafi et al., 2012; Hordeaux et al., 2015).

The unprecedented potential of self-complementary AAV9 for mediating widespread transgene expression in neuronal cells after systemic injection was recently demonstrated in both adult (Barkats, 2007; Duqué et al., 2009; Benkhelifa-Ziyyat et al., 2013) and neonatal animals (Barkats, 2007; Duqué et al., 2009; Foust et al., 2009; Benkhelifa-Ziyyat et al., 2013), including large animals such as cats (Barkats, 2007; Duqué et al., 2009) and NHPs (Bevan et al., 2011; Gray et al., 2011). This practical and non-invasive gene therapy approach has opened the way to first clinical trials in human, in particular for SMA type1 patients. Interestingly, recent studies showed that AAVrh10 also provided strong and widespread CNS transduction after intravenous administration in mice (Hu et al., 2010; Zhang et al., 2011; Mattar et al., 2013; Bucher et al., 2014; Hordeaux et al., 2015). In this study, we compared the capabilities of AAV9 and AAVrh10 at transducing different regions of the brain, the spinal cord and the PNS after intravenous injection in neonatal mice. As previously reported for AAV of serotypes 9 and rh10 by Cearley and Wolfe (2006), and with other serotypes by Davidson et al. (2000), variability was often observed between animals intravenously injected with the same serotype. However, the trends for a superiority of AAVrh10 transduction efficacy over AAV9 were evident in most CNS and PNS regions that we examined, with significant differences between the two vectors being found in the medulla, the cerebellum, the spinal cord and the sciatic nerves. Two main studies in neonatal mice have previously reported a greater transduction efficiency of AAVrh10 vs. AAV9 vectors in the brain following intravenous delivery (Hu et al., 2010; Zhang et al., 2011). As in our study, Zhang et al. showed in particular the superiority of AAVrh10 to transduce brain regions such as the hippocampus, the cerebellum or the medulla (Zhang et al., 2011). However, no difference between the two serotypes was reported in the spinal cord, in contrast to what we have demonstrated by western blot analysis of GFP protein levels in the whole spinal cord, quantification of GFP intensity levels in the dorsal spinal cord, and manual counting of the transduced MNs in the ventral spinal cord. Interestingly, our results showed that intravenous delivery of low dose AAVrh10 (3 × 10^13^ vg/kg) in neonatal mice led to the transduction of a number of MNs similar to that targeted with high dose AAV9 (10^14^ vg/kg). This finding suggested that using AAVrh10 could present potential for reducing the vector titer required for therapeutic translation to patients.

Of note, the superiority of AAVrh10 over AAV9 for MN transduction was mainly observed at low dose, the difference between the two vectors administered at high dose being only significant in the cervical spinal cord. Indeed, the increase of the vector titer induced a rise in AAV9 transduction efficiency, without further significant augmentation of that of AAVrh10. This finding is in accordance with the assumption that more AAVrh10 than AAV9 particles could transduce a single cell, without transducing an increased number of cells, as previously suggested in the comparative study of several AAV serotypes injected into the mouse brain (Cearley and Wolfe, 2006), and more recently, after infusion of the serotypes into the striatum of rats and pigs using convection-enhanced delivery (White et al., 2011). In the latter study, no greater distribution was induced by increasing the infusion titer of AAVrh10, in contrast to AAV9 whose distribution continued to rise (White et al., 2011). Likewise, we found the sciatic nerve to be preferentially transduced by AAVrh10, but only at low dose. Increasing the vector titer induced a marked increase of GFP intensity levels in whole sciatic nerve sections from AAV9 injected mice, without further rise when mice were injected with high dose AAVrh10, corroborating the results obtained in the spinal cord. Together, these results and those of the literature suggest that the superior transduction efficacy of AAVrh10 would be related to a greatest amount of particles entering nervous cells, rather than to a particular wide distribution of its receptors. Of note, the superiority of systemic AAVrh10 over AAV9 could also be dependent of genome conformation, since a study of Miyake et al., comparing several single-stranded AAVs (ssAAVs) intravenously delivered in neonatal mice, reported that transduction efficiencies of all vectors including ssAAVrh10 were low as compared to ssAAV9 (Miyake et al., 2011).

Differences between the serotype 9 and rh10 for CNS transduction efficiency could also be due to differential capabilities for entering nervous tissue following systemic delivery. Pathways by which AAV9 or AAVrh10 could enter the CNS could include transmigration or receptor mediated transcytosis across the endothelium of blood-brain barrier (BBB) and/or across the blood-cerebral spinal fluid barrier (blood-CSF barrier) at the choroid plexus as previously suggested (Barkats, 2007; Duqué et al., 2009) and previously assumed for HIV infection of the human brain (Falangola et al., 1995; Pereira and Nottet, 2000). As HIV viruses, AAV9 or AAVrh10 could also enter the CNS tissue at the level of the circumventricular organs (CVO), which are brain structures devoid of BBB thereby providing a possible site of infection (Johnson and Gross, 1993; Davson and Segal, 1996).

In view of the difference in the extent and intensity of transgene expression according to the brain structures, and of the gradient which was observed from the brain ventricles and adjacent regions to remote areas, our results are best in support of a preferential crossing at the choroid plexus. Indeed these structures were found to be robustly transduced in the 3rd and 4th ventricles, as well as neighboring parenchymal areas such as the hippocampus and the cerebellum. However, remote areas located far from the ventricles such as the cortex or the thalamus were also efficiently targeted, suggesting that other routes of entry into the CNS should also be taken, such as diffusion from the CVO. However, further experiments will be necessary for fully understanding the mechanism by which specific AAV serotype such as AAV9 and AAVrh10 enter into the brain and the spinal cord after systemic injection.

In addition to its high potential for CNS transduction, AAVrh10 was suggested to be an attractive alternative serotype to AAV9, particularly by the fact that it would be less prone to induce host serological immune response than AAV9 (Hordeaux et al., 2015). Indeed, humans should be less exposed to AAVrh10 since this vector is a rhesus monkey serotype, unlike the human AAV9 serotype. However, a recent study surprisingly reported a 59% IgG prevalence against AAVrh10 in humans, and 47% against AAV9 (Thwaite et al., 2015). Although most anti-AAVrh10 IgG were non-neutralizing (as anti-AAV9 IgG) their high prevalence in humans does not support the assumption of a particular immunological advantage of AAVrh10 over other serotypes. Moreover, antibody cross-recognition was also reported in humans, suggesting that a broad repertoire of preexisting antibodies would be able to react with non-human serotypes (Thwaite et al., 2015).

Although many studies have reported the high efficiency of AAVrh10 for CNS gene transfer, only a few compared AAV9 and rAAVrh10. This study suggests that, like AAV9, AAVrh10 holds promise for intravascular gene therapy of human CNS and PNS diseases affecting neurons, astrocytes, oligodendrocytes or Schwann cells. Our most significant finding was the superiority of AAVrh10 over AAV9 for MN transduction in neonatal mice, highlighting the particular potential of this serotype for SMA type1, a devastating disease affecting young children. Several brain regions were also reported to be affected in SMA type I patients, including the brainstem and the cerebellum (Harding et al., 2015), which were found highly transduced by serotype rh10. AAVrh10-mediated restoration of SMN in these brain areas could thus be important for gene therapy of SMA type 1. It should however be noted that the superiority of serotype rh10 over serotype 9 was mainly observed at low dose. The fact that AAVrh10 distribution was not improved by increasing the vector dose, whereas a dose-dependent increase of transduction efficiency was observed with AAV9, suggest differences in the transduction mechanisms of these two vectors which both present great interest for gene therapy of neurological diseases.

### Conflict of interest statement

The authors declare that the research was conducted in the absence of any commercial or financial relationships that could be construed as a potential conflict of interest.

## Supplementary material

Supplementary Figure 1 **Comparison of AAV9 and AAVrh10-mediated transduction of heart and liver**. Representative sections of **(A)** heart and **(B)** liver, treated for GFP immunofluorescence 30 days after delivery of AAV9 or AAVrh10 at low (a,b) and high (c,d) dose in neonatal mice (*n* = 4 per dose and per serotype). Green: GFP-immunopositive cells; panels at the bottom left corner: high magnification. (e) Quantification of the average GFP signal intensity/pixel on 12 immunolabelled heart (**A**,e) and liver (**B**,e) sections per mouse. Data are presented as mean of ± SEM of GFP fluorescence intensity/pixel (*n* = 4; Two-Way ANOVA variance analysis, Bonferroni *Post-hoc*-test: ^*^*p* < 0.05). Scale bars = **(A)** 1 mm; **(B)** 100 μm.
