# Supplementary figures and images for: Systemic AAVrh10 provides higher transgene expression than AAV9 in the brain and the spinal cord of neonatal mice
Source: Front Mol Neurosci. 2015 Jul 28;8:36. doi: 10.3389/fnmol.2015.00036 (PMC4516891; doi:10.3389/fnmol.2015.00036)

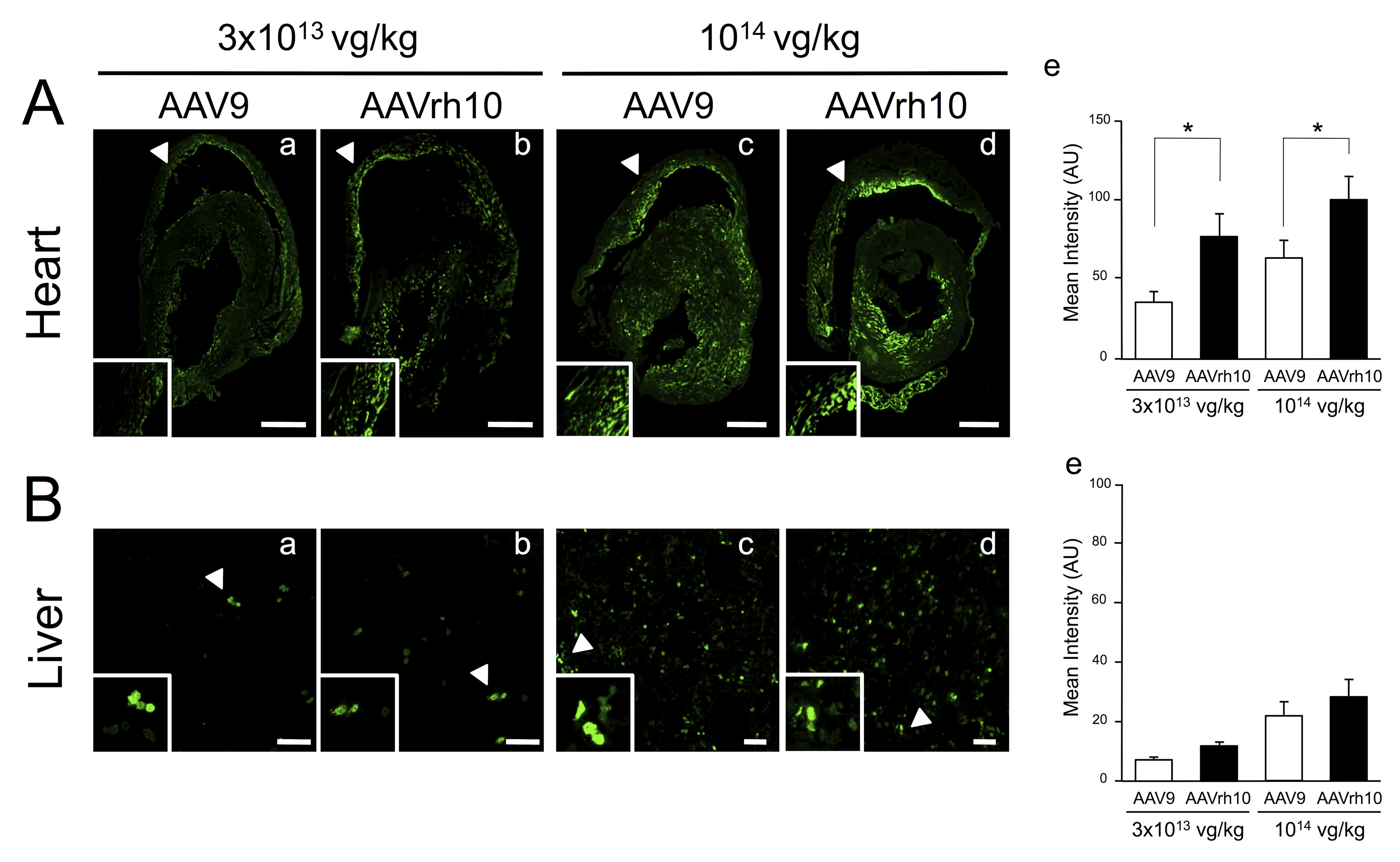

Supplement: Supplementary Figure 1 — Comparison of AAV9 and AAVrh10-mediated transduction of heart and liver. Representative sections of (A) heart and (B) liver, treated for GFP immunofluorescence 30 days after delivery of AAV9 or AAVrh10 at low (a,b) and high (c,d) dose in neonatal mice (n = 4 per dose and per serotype). Green: GFP-immunopositive cells; panels at the bottom left corner: high magnification. (e) Quantification of the average GFP signal intensity/pixel on 12 immunolabelled heart (A,e) and liver (B,e) sections per mouse. Data are presented as mean of ± SEM of GFP fluorescence intensity/pixel (n = 4; Two-Way ANOVA variance analysis, Bonferroni Post-hoc-test: *p < 0.05). Scale bars = (A) 1 mm; (B) 100 μm. [file Image1.JPEG]
